# The impact of a knowledge translation intervention employing educational outreach and a point-of-care reminder tool vs standard lay health worker training on tuberculosis treatment completion rates: study protocol for a cluster randomized controlled trial

**DOI:** 10.1186/s13063-016-1563-2

**Published:** 2016-09-07

**Authors:** Lisa M. Puchalski Ritchie, Monique van Lettow, Austine Makwakwa, Adrienne K. Chan, Jemila S. Hamid, Harry Kawonga, Alexandra L. C. Martiniuk, Michael J. Schull, Vanessa van Schoor, Merrick Zwarenstein, Jan Barnsley, Sharon E. Straus

**Affiliations:** 1 Department of Medicine, University of Toronto, Toronto, ON Canada; 2 Department of Emergency Medicine, University Health Network, Toronto, ON Canada; 3 Li Ka Shing Knowledge Institute, St. Michael’s Hospital, 30 Bond Street, Toronto, ON M5B 1W8 Canada; 4 Dignitas International, P.O. Box 1071, Zomba, Malawi; 5 Dalla Lana School of Public Health, University of Toronto, Toronto, ON Canada; 6 National Tuberculosis Control Program, Ministry of Health, P.O. Box 30377, Lilongwe, Malawi; 7 Sunnybrook Health Sciences Center, Toronto, ON Canada; 8 Dignitas International, 550 Queen Street East, Suite 335, Toronto, ON Canada; 9 George Institute for Global Health, Sydney, Australia; 10 The University of Sydney, Sydney, Australia; 11 Department of Family Medicine, Western University, London, ON Canada; 12 Department of Family Medicine, Schulich School of Medicine & Dentistry, Western University, 1151 Richmond Street, London, ON N6A 5C1 Canada; 13 Institute of Health Policy, Management and Evaluation, Dalla Lana School of Public Health, University of Toronto, 55 College Street, Suite 425, Toronto, ON M5T 3M6 Canada

**Keywords:** Lay health workers, Community health workers, Educational outreach, Reminders, Peer support network, TB, Tuberculosis, Cluster randomized trial

## Abstract

**Background:**

Despite availability of effective treatment, tuberculosis (TB) remains an important cause of morbidity and mortality globally, with low- and middle-income countries most affected. In many such settings, including Malawi, the high burden of disease and severe shortage of skilled healthcare workers has led to task-shifting of outpatient TB care to lay health workers (LHWs). LHWs improve access to healthcare and some outcomes, including TB completion rates, but lack of training and supervision limit their impact. The goals of this study are to improve TB care provided by LHWs in Malawi by refining, implementing, and evaluating a knowledge translation strategy designed to address a recognized gap in LHWs’ TB and job-specific knowledge and, through this, to improve patient outcomes.

**Methods/design:**

We are employing a mixed-methods design that includes a pragmatic cluster randomized controlled trial and a process evaluation using qualitative methods. Trial participants will include all health centers providing TB care in four districts in the South East Zone of Malawi. The intervention employs educational outreach, a point-of-care reminder tool, and a peer support network. The primary outcome is proportion of treatment successes, defined as the total of TB patients cured or completing treatment, with outcomes taken from Ministry of Health treatment records. With an alpha of 0.05, power of 0.80, a baseline treatment success of 0.80, intraclass correlation coefficient of 0.1 based on our pilot study, and an estimated 100 clusters (health centers providing TB care), a minimum of 6 patients per cluster is required to detect a clinically significant 0.10 increase in the proportion of treatment successes. Our process evaluation will include interviews with LHWs and patients, and a document analysis of LHW training logs, quarterly peer trainer meetings, and mentorship meeting notes. An estimated 10–15 LHWs and 10–15 patients will be required to reach saturation in each of 2 planned interview periods, for a total of 40–60 interview participants.

**Discussion:**

This study will directly inform the efforts of knowledge users within TB care and, through extension of the approach, other areas of care provided by LHWs in Malawi and other low- and middle-income countries.

**Trial registration:**

ClinicalTrials.gov NCT02533089. Registered 20 August 2015. Protocol Date/Version 29 May 2016/Version 2.

**Electronic supplementary material:**

The online version of this article (doi:10.1186/s13063-016-1563-2) contains supplementary material, which is available to authorized users.

## Background

The global shortage of skilled healthcare workers is estimated at 7.2 million, with the shortage most severe in Sub-Saharan Africa [1]. Task-shifting of less complex healthcare tasks to lay health workers (LHWs) is increasingly employed to address this shortage [2]. Despite the availability of effective treatment, tuberculosis (TB) remains an important cause of morbidity and mortality, with 9.6 million people falling ill and 1.5 million lives lost globally due to TB in 2014 [3]. The greatest proportion of new TB cases is in Africa, and over 95 % of TB deaths occur in low-income countries (LICs) [3]. In response to the high TB burden and severe healthcare worker shortages in these settings, outpatient TB care is among the tasks commonly shifted to LHWs.

LHWs are community members who have received some training but are not healthcare professionals [4]. Randomized trials show that LHWs improve access to basic health services and TB treatment outcomes by providing care and adherence support in the community [4, 5]. However, insufficient training and supervision are recognized barriers to LHWs’ effectiveness [5]. LHW training is typically conducted off-site [6], an approach that is expensive in both direct and opportunity costs due to disruption in care provision and thus limits training. Given their relative low cost and proven effectiveness, educational outreach and reminder knowledge translation (KT) strategies offer a promising solution to addressing LHW training needs by increasing incorporation of best evidence into LHW practice.

Malawi has among the lowest healthcare worker to population ratios, with 1.9 physicians and 28.3 nurses/midwives per 100,000 people [7]. In response to this severe health worker shortage, Malawi scaled up its LHW cadre to >10,000. As the primary providers of outpatient TB care, LHWs have a pivotal role in addressing the high TB burden in Malawi, with 17,723 new TB notifications in 2014 [8]. In spite of ongoing efforts, poor treatment adherence remains an important contributor to the high TB burden in Malawi, with treatment completion rates ranging from 58 % to 70 % in our recent study in Zomba District [9].

Despite their critical role, LHWs (termed *health surveillance assistants* [HSAs] in Malawi) in our recent study identified lack of disease- and job-specific training as the key barriers to their role as TB care providers [10]. The aims of this project are to address this knowledge-to-action gap by refining, implementing, and evaluating a KT strategy designed to improve LHW TB knowledge and counseling skills and, through this, to improve both TB care provided by LHWs and TB outcomes.

### Study objectives

Our goal is to improve TB care provided by LHWs in Malawi by refining, implementing, and evaluating a KT strategy designed to facilitate incorporation of evidence into routine LHW practice.

### Specific objectives

The specific objectives of our research are as follows:

1. Improve TB outcomes by implementing and evaluating a KT strategy developed and tested by our group to address an identified gap in care provided by LHWs in Malawi
2. Identify barriers to and facilitators of scalability and sustainability of this KT strategy, as well as its potential to address other gaps in care provided by LHWs

## Methods/design

### Study design

**Fig. 1:**
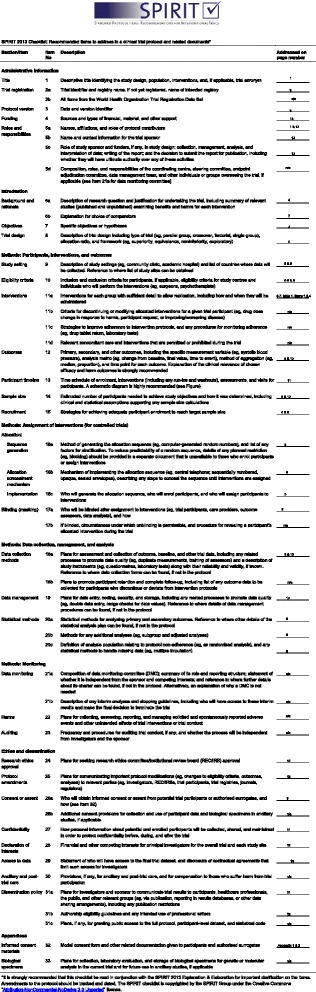
Standard protocol items recommended for intervention trials (SPIRIT) checklist

We will use a mixed-methods design that includes (1) a multicenter, pragmatic, cluster randomized controlled trial (RCT) to evaluate the effectiveness of the intervention and (2) a process evaluation employing qualitative methods including interviews with LHW participants and patients, as well as a document analysis of training logs, quarterly peer trainer meetings, and mentorship meeting notes, to gain an understanding of barriers to and facilitators of the implementation, scalability, and sustainability of the intervention. See Fig. 1 for details of where specific study elements are located in the protocol.

### Cluster RCT

#### Setting, participants, and randomization

Dignitas International (DI) works closely with the Malawi Ministry of Health (MOH) to support health system-strengthening and to build capacity among healthcare workers to improve clinical care and outcomes. This project will include all health centers (HCs) providing TB care among the 109 HCs in 4 of the 6 districts in which DI operates, excluding the district included in our preliminary study and an additional district that declined to participate. As TB care is provided at HCs on a rotating basis, patients receive care from several LHWs during treatment. Given this system of care, a cluster RCT (with allocation at the HC level) was chosen to prevent contamination. HCs will be randomly allocated in a 1:1 ratio based on a superiority framework using a computer-generated random number list prepared by a study team member without knowledge of the districts or HCs themselves, and they will be allocated by a second study team member. Once generated, the randomization information will be provided to a second study team member who will assign HCs as intervention or control sites by applying the random number list to the HC list provided by the district health offices. Randomization will be stratified by district, HC funding (MOH-funded vs non-MOH-funded), and HC designation as a priority site for support and mentorship. These stratification variables are chosen to address district-level variations in operationalization of TB policy and the potential for LHWs at priority sites to receive additional clinical training relevant to TB care.

#### Recruitment

Tuberculosis-focused lay health workers (TBLHWs) at participating HCs will be contacted by the DI district office in collaboration with the MOH district health offices. TBLHWs are general LHWs who receive 2 weeks of additional TB training and are responsible for TB care at the HC level. TBLHWs were selected as peer trainers by the MOH on the basis of their status and responsibilities as the local heads of TB care. In our previous work, we found TBLHWs to be effective in this role, as they were seen as experts by general LHWs, particularly after they were trained and had assumed the role of peer trainer. All LHWs routinely providing TB care will be eligible and invited to participate in the training, with refusal to participate being the only exclusion criterion. HCs and TBLHWs will be enrolled by the study coordinator (SC).

#### Inclusion and exclusion criteria

The trial will include all HCs providing TB care among the 109 HCs in 4 of 6 districts in which DI operates, excluding the district in which the pilot study was conducted and another district that declined to participate. HCs will be excluded if they do not routinely provide TB care.

#### KT intervention

**Fig. 2:**
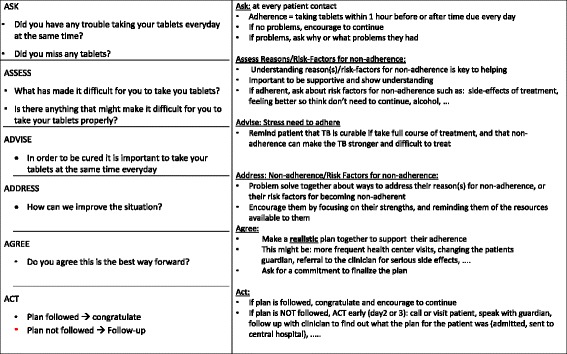
Lay Health Worker side of point-of-care tool, English version

**Fig. 3:**
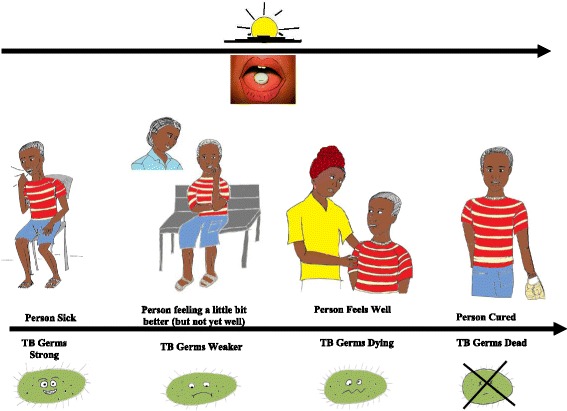
Patient side of point-of-care tool, English version

The current strategy builds on our earlier work, in which we identified a gap in LHW TB knowledge and job-specific training [9–11]. The multifaceted KT strategy will employ peer trainer-led educational outreach, a point-of-care reminder tool, and a peer mentoring network, chosen on the basis of evidence for the effectiveness of this approach with midlevel healthcare workers in South Africa [12–14], mapping of barriers to implementation identified through our formative qualitative study [10], and experience with and feedback from our prior studies [9, 11]. Improved patient TB knowledge and positive patient-provider interactions, two common barriers to adherence [15–18], are targeted through improved LHW skills in patient education and adherence counseling. Although evidence for communities of practice is poor [19], we include a peer mentorship network based on previous feedback from peer trainers to evaluate its potential role and cost implications. See Table 1 and Figs. 2 and 3 for detailed descriptions of the intervention and the point-of-care tool. The full manual is available upon request from the corresponding author.

**Table 1:** Description of the intervention

Details of intervention	Intervention group
Rationale/goals	The intervention was designed to target a recognized gap in TB care provided by LHWs by targeting two common barriers to adherence—patient disease understanding and patient-provider relationship—through improved LHW TB knowledge and skills in patient education and counseling.
Materials	The educational outreach component will use a combination of didactic and interactive techniques, including small- and large-group case-based discussions, role-playing to efficiently convey TB disease and treatment knowledge and patient education and counseling skills as well as to allow practice with the point-of-care tool and exchange of ideas between LHWs. Topics to be covered include TB transmission and treatment, risk factors for and consequences of poor adherence, the interaction of TB and HIV, treatment side effects and their management, and approaches to preventing and addressing nonadherence while maintaining a positive patient-provider relationship.
	The point-of-care tool (Figs. 2 and 3) is designed as a chart that can be folded and carried during field visits or stood on a desk for use during patient interactions. The LHW side of the tool provides a visual reminder designed to trigger an adherence discussion during patient encounters and provides clinical support for management of side effects and a constructive approach to addressing issues with adherence. The patient side uses simple pictorials to illustrate key messages used in patient education and adherence counseling. The tool was revised on the basis of feedback in our previous study, and usability was tested with two groups of LHWs, some new to the program and tool and some who had undergone the training and had used the original tool in the previous study in Zomba district. The manual is available upon request by contacting the corresponding author.
Procedures	Peer-led educational outreach sessions will occur on-site at participants’ base health center during regular work hours. Peer trainers will be asked to provide a minimum of eight sessions, each lasting a minimum of 1 h, over a 3-month period.
Intervention provider	TB-focused LHWs, who are general LHWs with 2 weeks of additional TB training and are responsible for TB care at the health center level, will be trained as peer trainers.
Method of delivery	Face to face
Location/context	Session will take place at the LHWs’ base health center during regular work hours.
Intensity	Eight sessions, each lasting a minimum of 1 h, over a 3-month period
Tailoring and modifications	Additional sessions as reinforcement opportunities, to train new staff, or as makeup sessions for staff who miss sessions will be left to the discretion of the peer trainers.
Fidelity	Fidelity will be assessed through peer trainers’ and general LHW participants’ self-report during mentor health center visits and through our process evaluation, which will include interviews with LHWs and a document analysis of LHW training logs, quarterly peer trainer meetings, and mentorship meeting notes.

*LHW* Lay health worker, *TB* Tuberculosis

The educational outreach component will employ on-site training led by the TBLHWs trained as peer trainers and delivered to small groups of general LHWs (typically five to ten) who provide TB care. Sessions will use both didactic and interactive techniques, including case-based learning and role-playing to convey TB and adherence knowledge and counseling skills and to allow for experiential learning through practice using the point-of-care tool, critical reflection, and exchange of ideas among LHWs. Topics will include TB transmission and treatment, common causes and consequences of nonadherence, and approaches to supporting adherence and addressing nonadherence while maintaining a positive patient-provider relationship. On the basis of learning from our previous studies [9, 11], two sessions will be added and the training period will be extended by 1 month to allow more time for each topic, and a reference manual will be provided in both English and Chichewa.

#### Training of peer trainers

Peer trainers will be trained over 4 days off-site by Lisa Puchalski Ritchie in English with the help of a sociolinguistic level interpreter [20]. Training will include content and techniques for peer training and supportive supervision. Peer trainers will be mentored by DI clinical staff during regular field visits to the HCs they support. On the basis of knowledge user feedback from our earlier work, development of a peer support network will be encouraged through quarterly in-person meetings that will bring together peer trainers in each district to share experiences, offer peer support, and provide an additional opportunity for mentorship from the implementation team. In addition, to encourage development of the peer support network, peer trainers will receive monthly phone credit throughout the study period, allowing them to call each other. If effective, this credit may be sustainable by the MOH, particularly during the initial rollout, which is the most challenging time for new peer trainers.

#### Training of LHWs

Peer trainers will provide a minimum of eight sessions, each lasting a minimum of 60 minutes, over a 3-month period. The sessions will be conducted on-site during regular work hours. All general LHWs who routinely provide TB care will be invited to participate. Extra sessions as reinforcement opportunities or to train new staff will be left to the discretion of the peer trainers. Training materials and certificates of completion will be provided. Incentives will not be provided, as training of general LHWs to assist with TB care is part of the TBLHWs’ job description, training will occur during regular work hours, and providing incentives would limit sustainability.

#### Point-of-care tool

Provided in Chichewa (see Figs. 2 and 3 for English version), the point-of-care tool is a two-sided flip chart that can be stood on a desk or carried during field visits. The patient side uses simple pictorials to illustrate a patient’s and TB bacterium’s course through treatment and acts as an aid for LHWs in providing patient education and adherence counseling. The LHW side provides a reminder to trigger an adherence discussion during patient interactions and supports side effect management and constructive approaches to addressing nonadherence. On the basis of our earlier work and heuristic testing, minor changes were made to the tool and a drug-dosing reference was added to it. Usability testing was then conducted with the tool to further refine it before implementation. This involved two cycles of iterative testing, each with three or four participants, including both previously trained LHWs and LHWs not previously trained with the original tool. As no appropriate patients were available at either HC during usability testing, participants were asked to role-play using the tool, with other HSAs or study team members playing the part of patients. “Patients” were provided with simulated cases based on real cases, with the goal of evaluating use of the tool with realistic patient examples, ranging from patients with newly diagnosed TB to complex and/or difficult cases. Detailed observation notes were taken by two observers. In addition, LHW participants were interviewed regarding their perceptions of and experiences with the point-of-care tool and asked to provide suggestions for improvement.

#### Control group

LHWs at control sites will receive usual training at the discretion of the HCs’ TBLHW. The content, format, and duration of the training varies considerably and ranges from a 1- to 2-h briefing on medication dispensing and form completion to a few days working alongside the TBLHWs as they provide patient care. LHWs will not be given access to the point-of-care tool or the peer network. Given the severe resource constraints of the Malawi healthcare system and the design of this intervention to specifically address an identified gap in care provided by LHWs to patients with TB, usual care was considered the most appropriate compactor against which to evaluate the effectiveness of the intervention in order to inform decision makers regarding scale-up and sustainability.

#### Informed consent

LHW participants in the educational intervention are HC personnel, who receive routine training and supervision. LHWs at intervention sites routinely involved in care of patients with TB will be encouraged but not required to attend training sessions. The educational intervention and point-of-care tool will be revised in collaboration with and approved by the National Tuberculosis Program (NTP) to ensure consistency with national TB treatment guidelines. As undergoing training is a routine expectation of HC staff and the training will be approved by the NTP, individual consent is not required for participation in the intervention.

#### Blinding

Given the nature of the intervention, blinding of participants is not possible.

#### Data collection

A digital copy will be made of TB registers of all participating districts at the end of the 1-year trial period. Data will be double-entered and verified by a data manager.

#### Outcomes

The primary trial outcome of interest is the proportion of patients with TB successfully treated (final value), defined according to the World Health Organization criteria [21] as the total number of patients cured and completing treatment. Secondary trial outcomes include the proportion of default cases (treatment interrupted for at least 2 consecutive months) and proportion of successes among cases with HIV coinfection. All outcomes will be assessed for 1 year following completion of LHW on-site peer-led training.

#### Sample size calculation

Although 109 HCs are available for participation in the 4 study districts, we expect that a small number do not routinely provide TB care. In addition, on the basis of our experience in the preliminary study, where several clusters were lost because of staff shortages necessitating transfer of TB cases or failure of HCs to accrue eligible TB cases in small, remote HCs, we have estimated the sample size for the present study conservatively as follows. With an alpha of 0.05, a power of 0.80, a baseline successful treatment completion of 0.80 at 1 year, an intraclass correlation coefficient of 0.1 based on our pilot study data, and an estimated 100 clusters (HCs that provide TB care), a minimum of 6 patients are required per cluster to detect a clinically significant 0.10 increase in the proportion of successful treatment completion.

#### Analysis plan

Summary statistics, including measures of central tendency and range, will be calculated and presented for each district, including number of HCs included, number of individuals receiving the intervention, number of LHWs trained at each site, baseline characteristics (proportion of pulmonary and nonpulmonary TB cases), proportion with TB-HIV coinfection, and TB outcomes across the trial arms by district.

The primary analysis will use multilevel modeling to compare proportion of treatment successes among the control and intervention groups, with analysis adjusted for correlation due to clustering and stratification. Multilevel modeling will also allow us to examine similarities and differences between and within districts (strata) and healthcare centers (clusters) in outcomes and for planned subgroup analysis. Analysis will be conducted on an as-randomized basis and performed using R statistical software.

### Process evaluation

#### Setting and participants

Interview participants will include LHWs who have received the intervention and patients and/or guardians who begin TB treatment on or after the trial start date and who are followed at a participating HC.

#### Inclusion and exclusion criteria

LHWs who have completed the educational outreach training and patients with TB of participating HCs who begin TB treatment during the trial period presenting for TB care on days the study research assistant (RA) is collecting data will be eligible for participation in the qualitative study. Exclusion criteria for interview participants include patients with TB who are younger than 18 years of age and unaccompanied by a parent or guardian, patients and/or guardians or LHWs who are unwilling or unable to give informed consent, patients who are not usually treated at the participating HC, and patients deemed by the local healthcare team to be too ill to participate.

#### Participant recruitment and informed consent

Two to four participants from each group (LHWs and patients) will be selected in each data collection period from each district and a maximum of two from any one HC. LHWs will be selected for interviews using mixed purposive sampling. A list of trained LHWs compiled by the peer trainers will provide the initial sampling frame. LHWs will be selected from among those on the list to represent the range of LHW characteristics in terms of gender, age, years of experience, and HC characteristics (rural vs urban). Three LHWs chosen to reflect the range of responses (positive to negative) in the first round of interviews will be selected to be interviewed at both study onset and conclusion. The study, SC, or RA will be introduced to the general LHWs by the peer trainers. LHWs will then be approached in person (or by phone if the selected LHW is not present on site at the time of the HC visit) by the SC or RA, who will use a recruitment script.

Convenience sampling will be used to select patients and/or guardians for interviews. Patients will be selected to represent the range of characteristics in terms of age, gender, and TB characteristics (new vs recurrent, pulmonary vs nonpulmonary). The SC or RA will attend HCs on days identified by HC staff as typically busy. The SC or RA will be introduced to patients by the LHWs working in the HC during HC visits. After being introduced, the SC or RA will approach patients in person, using a recruitment script.

Written informed consent will be obtained in person by the SC or RA prior to beginning the interview. In order to ensure participant understanding, in addition to providing the consent form in Chichewa, the SC or RA will read the consent form out loud. Participants will then be given an opportunity to read the consent form and to have any questions they may have answered by the study team. Once all questions are answered to the participants’ satisfaction, the participants will be asked if they wish to participate; if they agree, the form will be signed and witnessed. For patients under 18 years of age, consent will be obtained from the child’s parent or guardian using the same process, and assent will be obtained for children old enough to participate in interviews after parental consent has been obtained.

#### Outcomes

Process evaluation outcomes of interest include barriers to and facilitators of implementation, scalability, sustainability, and identification of potential program improvements.

#### Sample size calculation

Interviews will be conducted with LHWs and patients at 2 time points during the trial, with an estimated 10–15 participants from each group required each time to reach saturation and allow for sampling from all participating HCs, for a total of 40–60 participants.

#### Data collection

Interviews will be conducted with LHWs and patients at two time points to assess barriers to implementation and sustainability: in the first 3 months after training and in the last 3 months of the trial. Two or three LHWs will be interviewed both times in order to capture change within and across individuals over time. Participants will be interviewed by a trained RA fluent in English and Chichewa using a semistructured interview guide to ensure key areas of interest are addressed and to allow for emergence of novel themes. Interviews will be conducted in a private location (at or near the participants’ HC) at a time convenient to participants, with interviews expected to last 30–60 minutes. Interviews will be audio-recorded digitally using unique numeric identifiers only.

Training logs and quarterly peer trainer and mentorship meeting notes will be collected by the RA for analysis. No identifying data will be collected during the document review, with documents identified by unique numeric codes only.

#### Analysis plan

Interviews will be conducted by a trained Malawian SC or RA fluent in both English and Chichewa and functioning at the level of a sociolinguistic translator [20]. Interviews will be audio-taped, transcribed verbatim, and translated by an RA. Twenty percent of transcripts will be retranslated by a second RA as a quality check. Should discrepancies in conceptual equivalence be observed, all transcripts will be translated by a second interpreter, and discrepancies will be resolved by consensus. Interviews and training log entries will be analyzed using qualitative content analysis. Two study team members will read and code the transcripts, training logs, and meeting reports independently, with discrepancies resolved through consensus. NVivo 10 software (QSR International, Doncaster, Australia) will be used to code and organize data into themes. Themes will be sought within and across individuals, participant groups, and data collection periods to allow for assessment of change and emergence of themes over time. Results from qualitative data sources will be triangulated using the technique of integration, with data from all sources considered in detail to provide a more comprehensive understanding of the barriers to and facilitators of the sustainability and scalability of the intervention as well as use of the approach to address other gaps in care provided by LHWs.

#### Data management

The electronic copy of the recruitment list will be password-protected and stored on a secure server, maintained separate from the unique numeric identifier list, and accessible only by the principal investigators, an SC, and an RA. The recruitment list will be destroyed once the study is complete.

Digitized HC TB registers will be password-protected and stored on a secure server. Identifying data (name, village name, and TB number) will be used to verify records from double data entry only. Once verified, the name, village name, and TB number will be removed from the database, and records will be maintained using a unique identification number only. No personal identifiers will be collected from interview participants. Only unique numeric identifiers will be used for audio recordings and transcripts. Audio recordings will be destroyed once analysis is complete.

Consent forms, training logs, and quarterly peer trainer and mentorship meeting notes will be stored in a locked cabinet in a locked room and accessible only by the principal investigator, SC, and RA. No identifying data will be released at any time, with results reported in aggregate form only.

#### Participant timeline

**Fig. 4:**
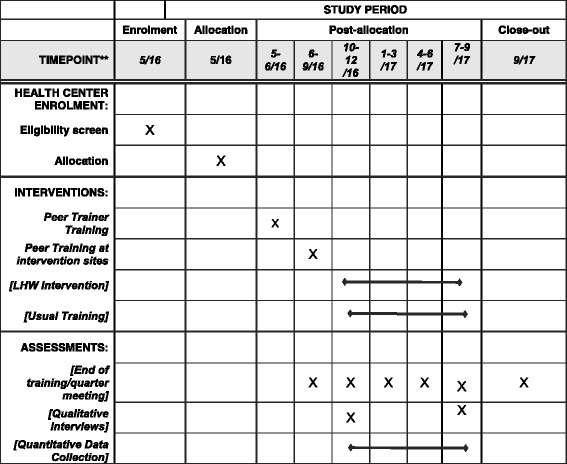
Standard Protocol Items: Recommendations for Interventional Trials (SPIRIT) checklist: schedule of enrollment, interventions, and assessments

Figure 4 shows the schedule of enrollment, interventions, and assessments.

#### Dissemination plan

Study findings will be submitted for peer-reviewed publication and for presentation at appropriate international conferences. In addition, study findings will be disseminated to participants and stakeholders through presentation at local meetings, and a one-page lay summary will be made available to participants and will be posted in the TB clinics of participating HCs.

## Discussion

Despite the availability of effective treatment, TB has a substantial impact on mortality in Malawi and other LICs. LHWs provide a potential solution to addressing the severe healthcare worker shortages and high TB burdens in these settings. However, to date, expansion of the LHW cadre and task-shifting of outpatient TB care in Malawi have failed to achieve the desired impact. The aim of our project is to refine, implement, and evaluate a KT intervention previously piloted in a single district in Malawi. The intervention is designed to improve uptake of evidence into routine practice of LHWs providing TB care in Malawi. Given the increasing role of LHWs in low- and middle-income countries, approaches to addressing knowledge gaps among LHWs through adequate training and supervision are essential to improving health outcomes.

The results of this study will inform the NTP efforts of the Malawi MOH, which is keen to implement the NTP nationally if proven effective. In addition, this project has the potential to generate principles that will inform programs to improve practice in other areas of care provided by LHWs in Malawi and in other LICs.

### Trial status

This study is currently in the early stages of implementation. Recruitment began on 6 May 2016.

## Additional files

Additional file 1: Letter of information and consent to participate in a research study - LHW version (English version). (DOCX 26 kb) Additional file 2: Letter of information and consent to participate in a research study - patient version (English version). (DOCX 28 kb)

## Abbreviations

DI: Dignitas International
HC: Health center
HSA: Health surveillance assistant
KT: Knowledge translation
LHW: Lay health worker
LIC: Low-income country
MOH: Ministry of Health
NTP: National Tuberculosis Control Program
RA: Research assistant
RCT: Randomized controlled trial
SC: Study coordinator
SPIRIT: Standard Protocol Items: Recommendations for Interventional Trials
TB: Tuberculosis
TBLHW: Tuberculosis-focused lay health worker
