# Supplementary material for: The impact of a knowledge translation intervention employing educational outreach and a point-of-care reminder tool vs standard lay health worker training on tuberculosis treatment completion rates: study protocol for a cluster randomized controlled trial
Source: Trials. 2016 Sep 7;17(1):439. doi: 10.1186/s13063-016-1563-2 (PMC5015212; doi:10.1186/s13063-016-1563-2)
Supplement: Additional file 2: — Letter of information and consent to participate in a research study - patient version (English version). (DOCX 28 kb) [file 13063_2016_1563_MOESM2_ESM.docx]

**Appendix F :** Letter of Information and Consent to Participate in a Research Study- Patient Version (English Version).

**Study Title:** Training Lay Healthcare Workers to optimize TB care and improve outcomes in Malawi

**Principal Investigators:** Lisa Puchalski Ritchie,MD, FRCPC, PhD; Monique van Lettow, PhD; Sharon Straus, MD, FRCPC, MSc.

**Study Contact Person:**

Lisa Puchalski Ritchie at 011 (416) 340-4800 ext 7183

Monique van Lettow 265 (0)1 525 420

Sharon Straus, 011 (416) 864-3068

You/your child are being asked to consider participating in a research study. Before agreeing to participate in this study, it is important that you read and understand the following description of the study, including the purpose, procedures, risks and benefits associated with this study. You should take as much time as you need to make your decision and ensure that all of your questions are answered by the study staff before signing the consent form. Participation in this study is voluntary, and you/your child may refuse to participate or withdraw from the study at any time. Choosing not to participate or withdrawing from the study will have no impact on the care you/your child receive from the TB clinic or health care center.

Purpose:

The purpose of this study is to understand your/your child’s experiences with the TB/HIV adherence intervention, and to identify what aspects of the intervention are helpful, what aspects are not helpful, any topics or issues that you/your child feel should be covered in the intervention that currently are not, any concerns you may have with the intervention, and any suggestions you/your child may have for improving the training or tool. The ultimate goal of the study is to provide information to allow us to improve the intervention and to understand how this type of intervention might be used to address HSA training needs in other clinical areas.

Procedure:

If you/your child agree to participate in this study, you/your child will be interviewed by a study team member. Interviews will last 30 to 60 minutes and will take place at a time and private location that are convenient to you. First you will be asked some basic questions about yourself/your child, such as your/your child’s age, gender, and how long you have been a TB patient. During the interview you/your child will be asked about your experiences with the intervention, your/your child’s perceptions of what aspects of the intervention you find helpful and not helpful, any topics or issues that you/your child feel should be covered in the intervention that are currently not, any concerns you/your child may have with the intervention, and any suggestions you/your child have for improving the training or tool. Interviews will be audio recorded. You/your child will be given a refreshment and 500 Kwacha stipend for your time.

Risks and Benefits of Study Participation:

The risks of participating in this study are small and may include distress from the questions. If any of the questions make you/your child uncomfortable you/your child may refuse to answer the question or withdraw from the study at any time, without penalty. There are no direct benefits to you/your child for participating in this study.

However, the information you/your child provide will be used to help improve the intervention which we hope will help HSAs working with TB patients and improve TB treatment outcomes for patients.

Confidentiality:

All persons involved in the study, including the study investigators and coordinators (hereby referred to as "study staff"), are committed to respecting your/your child’s privacy. No other persons will have access to your/your child’s personal health information without your consent, unless required by law.  The study personnel will make every effort to keep your/your child’s personal health information private and confidential in accordance with all applicable privacy legislations in Malawi and Ontario Canada.

Any personal identifying information (such as your/your child’s name) will be "de-identified" by replacing your/your child’s personal identifying information with a "unique code/number".  The study principal investigators are in control of the study unique code key which does not include your/your child’s name or other identifying information. The link between the study number and your/your child’s personal identity will be safeguarded by the principal investigators at St. Michael's Hospital and Dignitas International. You/your child will be identified only by a unique number. You/your child’s name will not be written be recorded on the audiotape and the audio recording will be accessible only to the principal investigators, study coordinator and a research assistant. Your/your child’s name will not be written in my notes or in any reports, publications, or presentations from this research. Your/your child’s data will not be shared with anyone except with your consent or as required by law.

To protect your/your child’s privacy, data will be password-protected and securely stored. In addition, access to records and data will be limited to authorized persons and transmission of the data will be secure. Hard copies of data will be stored in a locked cabinet in a locked room, at Dignitas International or the li ka shing knowledge institute in Toronto, Canada. Electronic records will be stored on encrypted USBs and or a secure server. The audio tape will be destroyed when the study is complete. The data and signed consent forms will be securely stored for 10 years and then destroyed.

Voluntary Participation:

Your/your child’s participation is voluntary, and you/your child may refuse to participate or withdraw at anytime, without penalty. If you/your child withdraw from the study the audiotape will be immediately destroyed. Participation, non-participation or withdrawal from the study will have no impact on the care you/your child receive from the TB clinic or health center. If you/your child withdraw your audiotape will be destroyed.

Questions about the study:

If you/your child have any questions about this study or your rights as a study participant, you may contact either of the individuals below at anytime:

You may contact the principal investigator Dr. Lisa Puchalski Ritchie at 011 (416) 340-4800 ext 7183 or Dr. Monique van Lettow 265 (0)1 525 420 or Dr. Sharon Straus 011(416) 864-3068

Alternatively if you have any questions regarding your/your child rights as a research participant, you may contact Dr. David Mazer, Chair, Research Ethics Board at 011 416-864-6060 ext. 2557 during business hours. Dr. Mazer is not part of the study team. Everything that you discuss will be kept confidential. Alternatively you may contact the National Health Sciences Committee Secretariat at 1 789 400, P.O. Box 30377, Lilongwe 3, Malawi.

Consent:

The research study has been explained to me, and my questions have been answered to my satisfaction. I have been informed of the alternatives to participation in this study. I have the right not to participate and the right to withdraw without affecting the quality of medical care at the TB clinic or health center for me and for other members of my family. As well, the potential harms and benefits (if any) of participating in this research study have been explained to me. I have been told that I have not waived my legal rights nor released the investigators, sponsors, or involved institutions from their legal and professional responsibilities. I know that I may ask now, or in the future, any questions I have about the study. I have been told that records relating to me and my care will be kept confidential and that no information will be disclosed without my permission unless required by law. The consent form has been read to me and I have been given sufficient time to read the above information. I consent to participate. I have been told I will be given a signed copy of this consent form.

_________________________________     ________________________      __________________

Participant/Guardian Name (Please Print)  Participant/Guardian Signature  Date

I confirm that I have explained the nature and purpose of the study to the participant/guardian   named above and answered all questions and that I have attained assent from any participants under 18 years of age old enough to participate in the interview

__________________________       __________________________        ___________________

Name of Person Obtaining Consent  Signature  Date

_______________________________________________________ ______________________

Name and signature of Witness for patients unable to sign Date
